# Innovative Vancomycin-Loaded Hydrogel-Based Systems – New Opportunities for the Antibiotic Therapy

**DOI:** 10.2147/IJN.S443051

**Published:** 2024-05-03

**Authors:** Aleksandra Florczyk, Aleksandra Krajcer, Kinga Wójcik, Joanna Lewandowska-Łańcucka

**Affiliations:** 1 Faculty of Chemistry, Jagiellonian University, Kraków, Poland; 2 Doctoral School of Exact and Natural Sciences, Jagiellonian University, Kraków, Poland; 3 Faculty of Biochemistry, Biophysics and Biotechnology, Jagiellonian University, Kraków, Poland

**Keywords:** vancomycin, hydrogels, chitosan-based capsules, biopolymers, delivery systems

## Abstract

**Purpose:**

Surgical site infections pose a significant challenge for medical services. Systemic antibiotics may be insufficient in preventing bacterial biofilm development. With the local administration of antibiotics, it is easier to minimize possible complications, achieve drugs’ higher concentration at the injured site, as well as provide their more sustained release. Therefore, the main objective of the proposed herein studies was the fabrication and characterization of innovative hydrogel-based composites for local vancomycin (VAN) therapy.

**Methods:**

Presented systems are composed of ionically gelled chitosan particles loaded with vancomycin, embedded into biomimetic collagen/chitosan/hyaluronic acid-based hydrogels crosslinked with genipin and freeze-dried to serve in a flake/disc-like form. VAN-loaded carriers were characterized for their size, stability, and encapsulation efficiency (EE) using dynamic light scattering technique, zeta potential measurements, and UV–Vis spectroscopy, respectively. The synthesized composites were tested in terms of their physicochemical and biological features.

**Results:**

Spherical structures with sizes of about 200 nm and encapsulation efficiencies reaching values of approximately 60% were obtained. It was found that the resulting particles exhibit stability over time. The antibacterial activity of the developed materials against *Staphylococcus aureus* was established. Moreover, in vitro cell culture study revealed that the surfaces of all prepared systems are biocompatible as they supported the proliferation and adhesion of the model MG-63 cells. In addition, we have demonstrated significantly prolonged VAN release while minimizing the initial burst effect for the composites compared to bare nanoparticles and verified their desired physicochemical features during swellability, and degradation experiments.

**Conclusion:**

It is expected that the developed herein system will enable direct delivery of the antibiotic at an exposed to infections surgical site, providing drugs sustained release and thus will reduce the risk of systemic toxicity. This strategy would both inhibit biofilm formation and accelerate the healing process.

## Graphical Abstract

**Figure:**
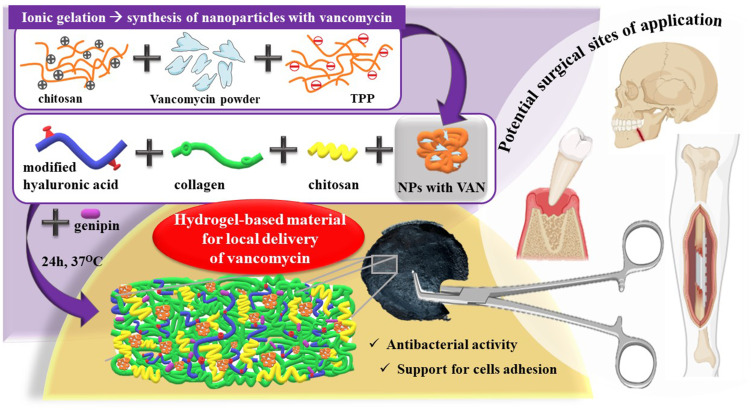

## Introduction

Bacterial infections (neuroinfections/bone infections) that can occur during implantation/medical surgeries are a serious problem for health care systems that cause important socioeconomic implications.^1^ Implant-related infections often lead to long-term pain for the patient, loss of functionality, and huge treatment costs. Therefore, early infection control is of key importance to prevent the occurrence of its severe course and reduce healthcare expenses.^2^ When tissue is infected, the antibiotic administration is required and the main challenge then is to reach the targeted site with a pharmaceutical agent, which is difficult to achieve in conventional therapies.^3^,^4^ Besides, the pharmacokinetics of antibiotics in terms of short half-life and systemic toxicity limit the application of high drugs’ doses.^5^ To address this issue, local or site-specific antibiotic drug delivery systems (DDS) based on various types of inorganic/organic materials have been developed.^6–8^ Compared with traditional systemic administration, they can effectively raise the drug concentrations within the infected sites, ensure greater control over the toxicity of dose and thus the risk of antibiotic resistance is also reduced.^9^

Vancomycin (VAN), a broad-spectrum glycopeptide antibiotic, is a comprehensive drug for bacterial infections treatment, especially gram-positive ones and those due to Methicillin-Resistant Staphylococcus aureus (MRSA).^10^ MRSA is the most prevalent multi-drug resistant pathogen in the world, being one of the main sources of infection in patients with a history of hospitalization.^11^ VAN is applied particularly in the treatment and prophylaxis of staphylococcal infections including those caused by MRSA (eg diabetic foot ulcer infections) and one of the drugs of choice against pathogens for bone infections (osteomyelitis).^12^,^13^ Furthermore, VAN is also recommended for the treatment of brain abscesses in postoperative neurosurgical patients.^14^ Successful treatment requires a long-term (usually 4−8 weeks) hospitalization, resulting in high expenses and lower quality of life for patients. Long-term intravenous VAN administration may cause serious toxicity (nephrotoxicity, ototoxicity, and hypersensitivity reactions); therefore, direct local VAN delivery via biodegradable devices is also highly desired.^14^,^15^ In this respect, natural biodegradable polymers such as chitosan, sodium alginate, and hyaluronic acid are widely used for particle-based drug delivery systems designing.^16–18^ However, the main difficulty associated with particles as DDS is their “burst release”, causing the release of a substantial amount of the drug within a short time, decreasing thus the effectiveness of therapy. Moreover, these initial high release rates may lead to supraphysiological levels of drug concentrations near or above the toxic level in vivo.^19^ It was reported that by immobilizing particles in the polymeric network, this phenomenon can be efficiently limited, and simultaneously the release time/rate might be prolonged.^20^

Therefore, to address this issue, we have developed the innovative hydrogel-based composites for the local VAN therapy. Presented herein, the systems are composed of VAN-loaded-chitosan-based particles embedded into biomimetic collagen/chitosan/hyaluronic acid-based hydrogels crosslinked with genipin and freeze-dried to serve in a solid flake/disc-like form.^21^ Hydrogel-based materials have been recently exploited in various biomedical fields, including drug delivery, tissue engineering, and regenerative medicine.^22^ Importantly, employing degradable polymers the absorbable/biodegradable systems might be fabricated, and thus there is no need for their surgical removal. However, the intrinsic permeability of the hydrogel matrix might noticeably limit the sustained and controlled delivery of drugs using such macromolecular carriers. Therefore, to enhance the therapeutic effect, eliminate the uncontrolled leakage and burst release, we have developed systems in which the VAN was first encapsulated in chitosan-based microcapsules and then dispersed within the hydrogel matrix. It is expected that such a designed system will enable direct delivery of the active substance to the disease environment during implantation/medical surgeries, thus reducing the systemic toxicity and providing its control/sustained release. The resulting materials were characterized for their physicochemical and biological properties, with particular emphasis on the VAN encapsulation efficiency. The swelling ability, degradation, antibacterial activity, and microstructure of the resulting polymeric films were also evaluated. Furthermore, preliminary biocompatibility in vitro experiments were performed using MG-63 cells as a model. All these parameters are crucial when considering the potential biomedical applications. To the best of our knowledge, such a system for VAN delivery has not been presented in the literature yet.

## Experimental Part

### Materials

Sodium tripolyphosphate (TPP, Sigma-Aldrich, Germany), vancomycin hydrochloride (VAN, Sigma-Aldrich, China), FITC-labeled vancomycin (Sigma-Aldrich, Israel), and phosphate buffer saline (PBS, Sigma-Aldrich, USA). Collagen (Col) type I rat tail (3.89 mg/mL solution in 0.02 N acetic acid Corning, USA), chitosan (Chit) (low molecular weight, TCI, Tokyo, Japan), hyaluronic acid (HA) (M_w_ ~ (1.5–1.8) × 10^6^ Da, Sigma-Aldrich, Czech Republic) was functionalized with lysine (HA_mod_) according to the procedure described by us earlier^21^ (substitution degree about 17% was calculated based on elementary analysis and ^1^HNMR spectroscopy), genipin (Challenge Bioproducts Co., 98%, Taiwan), Dulbecco’s Modified Eagle Medium (DMEM, Sigma-Aldrich, UK), penicillin–streptomycin solution (10.00 units/mL, HyClone, USA), fetal bovine serum (FBS, HyClone, USA), trypsin (HyClone, USA), Alamar Blue reagent (Invitrogen, USA), glutaraldehyde solution (Sigma-Aldrich, USA), hexamethyldisilazane reagent grade (HMDS, Sigma-Aldrich, Germany), osteoblasts-like cells: MG-63 (ATCC^®^ CRL-1427™, Manassas, USA) (Organism: Homo sapiens; Tissue: bone; Disease: osteosarcoma). Solvents: Acetic acid 99.5–99.9% (POCH, Gliwice, Poland) and ethyl alcohol 96% (STANLAB, Lublin, Poland). Materials: Vivaspin 2 mL, 5000 MWCO PES membrane (Sartorius, UK), SnakeSkin dialysis tubing 3.5K MWCO (Thermo scientific, USA).

### Methods

#### Vancomycin-Loaded Chitosan Particles Preparation

Chitosan nanoparticles were fabricated by the ionotropic gelation method using tripolyphosphate sodium (TPP), with some modifications to the procedures reported in the literature.^23^ The chitosan solution at a concentration of 2 mg/mL was prepared in 0.1M aqueous acetate buffer solution (pH=4.9). The dissolved polymer was next sonicated in an ultrasonic bath by setting the parameters to a pulse mode of 30 minutes and then 10 minutes of continuous mode and filtered through a 0.45 μm filter. VAN powder (9 mg) was dissolved in 15 mL of the filtered chitosan solution. The entire setup was heated to 60°C using a water bath. Then, 4.5 mL of 2 mg/mL TPP in filtered distilled water (stored at approximately 4°C) was added dropwise to the prepared system. The mixture was stirred for 24 hours at 250 rpm on a magnetic stirrer. VAN-loaded particles were separated from the suspension medium by centrifugation. For this purpose, 2 mL of the obtained particle suspension was added to an ultrafiltration tube (Vivaspin 5000 MWCO) and then centrifuged at 7000 rpm (10°C) for 25 minutes. Two fractions, concentrate and filtrate, were obtained and diluted to 2 mL with 0.1M acetate buffer. The concentrate fraction was collected for further use.

#### Preparation of Model Chitosan Particles Loaded with Fluorescently Labeled Vancomycin

The chitosan particles with encapsulated fluorescently labeled vancomycin were prepared following the method described in paragraph “Vancomycin-loaded chitosan particles preparation”. However, instead of pure VAN, FITC-labeled vancomycin at a concentration of 12.5 μg/mL in chitosan solution was used.

#### Preparation of Hydrogels with Embedded Vancomycin-Loaded Chitosan Particles

Hydrogel-based systems composed of collagen (Col), chitosan (Chit), lysine-modified hyaluronic acid (HA_mod_) (Col/Chit/HA_mod_) and vancomycin-loaded chitosan particles were obtained according to a modified procedure described by us previously.^21^ Briefly, the appropriate polymer solutions: 540 μL of Col, 84 μL of Chit (10 mg/mL in 1% acetic acid) and 126 μL of HA_mod_ (10 mg/mL in 10x PBS buffer) were mixed. Next, 200 μL of particles dispersion of the selected VAN concentration and 50 μL of genipin solution (18 mg/mL solution in 96% ethanol) were added. Obtained mixtures were vigorously vortexed and incubated at 37°C until gel formation (about 24 hours). After this time, the cross-linked material was rinsed three times with deionized water and then frozen and lyophilized in an Alpha 1–2 LDPLUS lyophilizer. The collagen/chitosan/modified hyaluronic acid weight ratio in the resulting systems was 50:20:30 (Col/Chit/HA_mod_). Hybrids denoted as HC1, HC2, and HC3, contained 0.09; 0.18, and 0.36 mg/mL VAN in chitosan solution, respectively. Hydrogel with chitosan-based particles without loaded VAN serves as a control sample.

### Characterization of Vancomycin-Loaded Chitosan Particles

#### Light Scattering and Zeta Potential Measurements

The resulting particles were characterized in terms of their hydrodynamic diameter and zeta potential values using a Malvern Nano ZS light-scattering apparatus (Malvern Instrument Ltd., Worcestershire, UK). The sample of solutions was illuminated by a 633 nm laser, and the intensity of light scattered at an angle of 173° was measured by an avalanche photodiode. Polystyrene cuvettes were used for DLS measurements. Chitosan particles were diluted twice with an acetate buffer solution (pH=4.9) at a concentration of 0.1M immediately prior to measurement. The z-averaged hydrodynamic mean diameters and dispersity index (DI) of the samples were calculated using the software provided by Malvern. The ξ-potential of particles was measured using laser Doppler Velocimetry (LDV) in a capillary cuvette. Each value was obtained as an average of three measurements and cycles were performed for all systems tested. In all cases, the measurement uncertainty is the standard deviation.

#### Nanoparticle Tracking Analysis (NTA)

The vancomycin-loaded chitosan particles’ concentration was determined using a NanoSight NS300 instrument equipped with an sCMOS camera and a 488 nm blue laser. Data were calculated with the NTA software (3.4 Build 3.4.4). The size distribution was measured at camera level 10 (shutter: 696, gain: 73). A single experiment consisted of five repetitions, each at 25 frames/second.

#### Turbiscan Measurements

The stability of vancomycin-loaded chitosan particles was monitored using the optical characterization method with Turbiscan Classic 2 (Formulation). The analyzed dispersion (5 mL) was placed in a cylindrical glass cell, and then the transmission light intensity was measured by scanning the sample along its height every 2h for a period of 7 days at room temperature. The data obtained were analyzed using the TurbiSoft software. Dispersion stability was determined with the Turbiscan Stability Index.^24^

$$TSI = \mathop \sum \limits_J \left| {sca{n_{ref}}({h_j}) - sca{n_i}({h_j})} \right|$$

Where $sca{n_{ref}}$ is an initial transmission value at a particular time, $sca{n_i}$ is a transmission value at the same time when $({h_j})$ is a certain height in the measuring cell.

#### Determination of Encapsulation Efficiency

To determine the encapsulation efficiency of VAN loaded in the Chit-NPs, the fractions obtained using Vivaspin 5000 MWCO were subjected to UV–Vis measurements on a Varian Cary 50 spectrometer. The excess of free VAN has been removed by Vivaspin concentrators. UV–Vis spectra of the initial samples (before centrifugation) and those after centrifugation (concentrate and filtrate) were recorded. Measurements were made in the range of 200–500 nm, and the reference solution was 0.1M acetate buffer solution. The drug loaded in the chitosan particles was calculated based on the calibration curve, prepared by measuring the UV–Vis absorbance of the known concentration of VAN in the range of 0.008–0.250 mg/mL. The encapsulation efficiency (*EE*) of VAN in chitosan particles was calculated according to the following equation:^25^

$$EE = {{{c_t} - {c_f}} \over {{c_t}}}.\;100\rm\% $$

Where ${c_t}$ presents the total concentration of VAN used for the preparation of particles and ${c_f}$ represents the concentration of unencapsulated drug present in the filtrate. The estimated EE for nanoparticles with the highest content of VAN was 59.08 ± 2.85%.

### Characterization of Hydrogels with Embedded Vancomycin-Loaded Chitosan

#### Degradation Study

The degradation process of lyophilized hydrogels under physiological conditions (PBS (pH=7.4), 37°C) was investigated. For this purpose, a series of hydrogels (three repetitions for each type) were first incubated for 2h in deionized water, weighed and obtained values were taken as 100% of the total mass of hydrogels (*M*_0_). The hydrogels were next incubated at 37°C in PBS buffer solution, with gentle shaking. At the defined time points (4, 24, 48, 72, 168, 240, 362, and 700 hours of incubation), the materials were weighed and then a fresh amount of PBS buffer was added to the system. Weight changes during the experiment were calculated according to the following equation.^26^

$$remaining\,weight \left({wt\rm\%} \right) = {{{M_t}} \over {{M_0}}}.\;100\rm\%$$

#### Swelling Evaluation

The developed lyophilized hydrogels’ swellability was studied under physiological conditions by materials incubation at 37^°^C in PBS buffer, with gentle shaking for 24h. After that, PBS buffer was removed, the hydrogels were rinsed three times with deionized water, lyophilized and weighed (${W_s}$). The materials were then lyophilized and weighed again (${W_d}$). The following formula was used to calculate the swelling ratio (*SR*):^26^

$$SR = {{{W_s} - {W_d}} \over {{W_d}}}.\;100\rm\%$$

For each series of samples, the measurements were carried out in triplicates and the results are presented as the averages ±SD.

#### The model release studies of fluorescently labeled vancomycin (VAN-FITC)

Hydrogels embedded with FITC-labeled vancomycin-loaded chitosan particles were subjected to drug release evaluation. Fluorescently labeled VAN allows the release kinetics to be followed with greater sensitivity. The lyophilized samples were placed in a 24-well plate and 0.5 mL of PBS buffer was added to each well and incubated at 37°C with gentle shaking being protected against light. Such an amount of buffer was used to provide the sink condition. At certain time points (30 min, 1h, 2h, 5h, 8h, 24h, 48h, 72h, 168h, 240h, 336h, 504h, 840h, 1009h, 1081h) all medium was collected and replaced with a fresh PBS buffer. The same experiment was performed for the references that were chitosan particles loaded with VAN-FTIC. In this case, we used the 3.5K MWCO dialysis tube with 3 mL dispersion, which was placed in a beaker containing a release medium (22.5 mL of PBS, pH=7.4), sealed, and shaken at constant speed at 37°C. At the time points (30 min, 1h, 2h, 5h, 8h, 24h, 48h, 72h, 168h, 240h, 336h), 2 mL from the release medium was collected (2 mL) and stored until further tests. An equal amount of fresh PBS was added to the solution. To determine the amount of VAN, the spectrofluorometric method was used; the fluorescence intensity at 518 nm was monitored, and then the value obtained was correlated with the calibration curve. The instrument (Hitachi F-7100 FL Spectrophotometer) parameters were set as follows: EX WL 480 nm, EM Start WL 490, EM End WL 600 nm, scan speed 240 nm/min, EX and EM Slit 5.0 nm, PMT Voltage 400V. The experiments were performed in triplicate, and the results are presented as averages with standard deviation.

#### In vitro Osteoblasts Culture

For the biological tests, a series of hydrogel-based systems (three samples for each type of materials) were prepared in a 96-well plate, sterilized with UV radiation, and incubated with the addition of a cell culture medium without serum for about 1h at 37°C. Before cell culture, the medium was removed, and the MG-63 cell line was seeded in the plate at a density of 2 × 10^4^ cells/cm^2^. Medium containing 90 vol% DMEM (supplemented with 1 vol% of penicillin–streptomycin solution) and 10 vol% of serum was used. After 1 and 3 days of culture, the cell viability was studied using Alamar Blue (AB) assay according to the procedure employed by us previously.^27^ For cells’ distribution and morphology analysis after 3 days of MG-63 cells culturing materials were fixed and dehydrated using protocol from.^26^ The samples were next stuck on carbon tape, sputtered with gold, and SEM analysis was conducted using a cold field scanning electron microscope HITACHI S-4700 equipped with a NORAN Vantage energy dispersion spectrometer.

#### Antibacterial Activity Assessment

A series of hydrogel materials were subjected to antibacterial activity against *Staphylococcus aureus* (ATCC 25923) (Gram-positive bacteria). The composites (containing 7.29, 3.65, and 1.82 μg of VAN, respectively) were prepared in a 96-well plate and sterilized with UV radiation for 20 min. *Staphylococcus aureus* was grown in Mueller–Hinton (MH) agar medium until the logarithmic phase of growth was reached. After that, the bacteria were spread on the surface of (MH) agar medium in Petri dishes with a concentration in the range of 1.77 · 10^5^ - 8.3 · 10^6^˙ cells per 1 cm^2^ of agar surface. Hydrogel discs were subsequently placed on the surface of such prepared dishes and 5–10 μL of acetate buffer was gently placed on them. In the case of particles and VAN solution (in acetate buffer), pure paper discs were first placed on the Petri dishes prepared as described above and next 20 μL of particles dispersion/VAN solution was placed on discs. The control sample for VAN solution was acetate buffer. All plates were left at room temperature for 4h, and next placed in an incubator at 37°C throughout the night.

### Statistical Analysis

The experiments were repeated three times, and the results were expressed as mean ± standard deviation. Statistical significance was calculated using Student’s *t*-test. A comparison between the two means was analyzed with the statistical significance level set at p < 0.05.

## Results and Discussion

### Physicochemical Characterization of Vancomycin-Loaded Chitosan Particles

The sample with the highest content of VAN was characterized in terms of size distribution by means of DLS, NTA, and SEM techniques. Moreover, the zeta potential of the particles, their stability over time and VAN encapsulation efficiency were also determined.

### DLS/Zeta Potential and NTA Measurements

**Figure 1:**
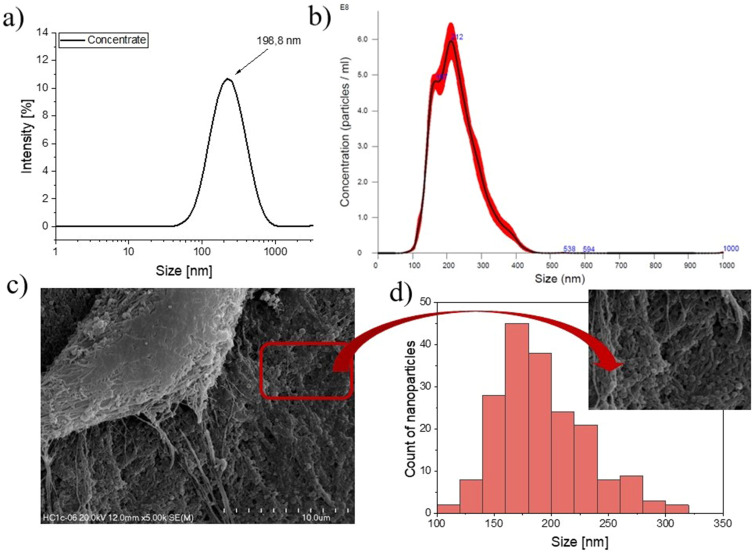
VAN-loaded-Chit-NPs-concentrate size characterization obtained by (**a**) DLS (intensity based), (**b**) NTA (Averaged FTLA Concentration/Size), (**c**) SEM micrographs and (**d**) size distribution histogram based on SEM studies.

In Table 1, the hydrodynamic diameter, DI, and zeta potential values for VAN-loaded-Chit-NPs and control sample (Chit-NPs) before and after (concentrate) centrifugation in Vivaspin concentrators are presented. In Figure 1a the size distribution profiles of VAN-loaded-Chit-NPs-concentrate determined from DLS are depicted. Comparing the results for the control sample and VAN-loaded particles, it can be noticed that the mean hydrodynamic diameter (d_z_) increased slightly after loading, with the values changing from about 226 nm to 246 nm, respectively. As expected, the size and DI measured for both systems after centrifugation (concentrate samples) went down and reached the hydrodynamic diameters and DI values about d_z_=158 nm; DI=0.205 for Chit-NPs and d_z_=203 nm; DI=0.258 for VAN-loaded-Chit-NPs, respectively. We have also determined the zeta potential of the obtained particles. The zeta potential, defined as the electrokinetic potential calculated based on the electrophoretic mobility of particles, allows the determination of their stability. The theory of stability states that the durability of colloids depends on the balance between van der Waals attractive forces and electrostatic repulsive forces.^28^ It is known that the pH and ionic strength of the solution strongly affect the value of objects’ zeta potential; therefore, the measurements were performed under the same pH conditions (pH=4.9). The zeta potential value obtained for particles loaded with VAN after centrifugation (26.3 ± 2.4) is at the same level as for the control formulation (25.0 ± 2.4) (see Table 1). This observation can be explained assuming that the VAN molecules are indeed located inside the chitosan-based particles. In addition, the revealed substantial differences in particles’ size confirmed the incorporation of a significant amount of antibiotics into the carriers. Encapsulation efficiency for nanoparticles with the highest content of VAN calculated based on the UV-Vis spectroscopy was 59.08 ± 2.85%. A significant amount of encapsulated drug affected the geometry of the structure, thereby altering particle size as demonstrated by the DLS measurements.

**Table 1:** Summary of Dynamic Light Scattering and Zeta Potential (at pH=4.9) measurements carried out for Chit-NPs and VAN-Loaded-Chit-NPs, respectively

Type of the Sample	Hydrodynamic Diameter [nm]	DI	Zeta Potential [mV]
Chit-NPs (Control)	226 ± 2	0.254	25.1 ± 2.6
Chit-NPs-concentrate	158 ± 2	0.205	25.0 ± 2.4
VAN-loaded-Chit-NPs	245 ± 3	0.283	28.0 ± 1.3
VAN-loaded-Chit-NPs-concentrate	203 ± 1	0.258	26.3 ± 2.4

The VAN-loaded-Chit-NPs-concentrate sample was additionally characterized using nanoparticle tracking analysis (NTA). The direct real-time visualization of subjects under constant flow exhibits high sensitivity and each particle undergoes separate picturing and analysis, enhancing thus the precision of the technique. Moreover, using NTA it is also possible to assess the concentration of objects present in the sample. Figure 1b shows the size distribution profiles determined from NTA presenting the relationship between formulation size and particle concentration in the dispersion. As can be noticed, the maximum peak was revealed for objects with sizes around 212 nm. The experimentally obtained concentration for sample C3 is 8.61 · 10^10^ ± 2.73 · 10^9^ particles/mL.

Finally, to visualize the developed particles, they were analyzed being embedded within the Col/Chit/HA_mod_ hydrogel by means of SEM. Based on the obtained SEM images (Figure 1c) and taking into account the sizes of 190 objects a histogram was created (Figure 1d). As can be seen in Figure 1d the highest number of particles with diameters of about 170 nm was found. Overall, by comparing three techniques used for measuring the sizes of the obtained structures, it was observed that the DLS and NTA results are in good agreement. However, when using SEM micrographs and ImageJ software, the objects’ diameters were lower. The discrepancy between the particle sizes obtained by DLS/NTA and SEM resulted from the differences in these experimental techniques. The DLS/NTA methods measure the mean hydrodynamic diameter, that is, the size of the particle together with the layer of ions and solvent molecules surrounding it. While SEM informs about the size and shape of the objects without the solvating shell removed previously by lyophilization.

### Stability Over Time

**Figure 2:**
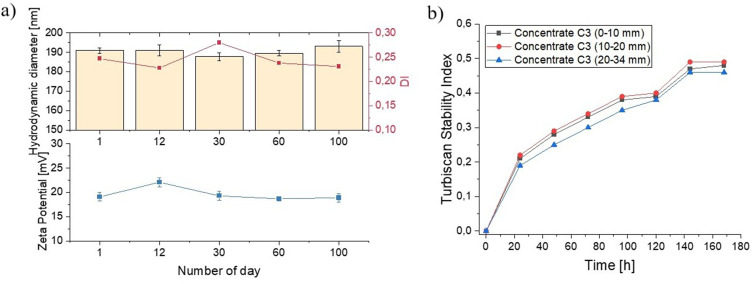
Stability over time for vancomycin–loaded chitosan nanoparticles-concentrate (VAN-Chit-NPs-concentrate): (**a**) size and zeta potential analysis by dynamic light scattering, (**b**) Turbiscan Stability Index parameter during a 7-day experiment.

NPs designed for potential biological applications are generally intended to exhibit behavior similar to macromolecules dissolved in solution. Therefore, undesirable phenomena such as aggregation, disintegration, or changes in morphology/shape may occur.^29^ To develop the final composites in the form of vancomycin-loaded chitosan particles embedded within a hydrogel matrix, it was of great importance to investigate the long-term stability of the resulting formulations. Thus, we have performed experiments in which the particles’ dispersions were stored in the refrigerator and were analysed at several time points to reveal the possible changes in the size and the zeta potential. The obtained results are depicted in Figure 2a. No significant differences in the sizes of the objects were found during storage. The hydrodynamic diameter of the particles oscillates around 200 nm, and the dispersity index is below 0.3, indicating a relatively narrow size distribution and the lack of aggregation. The measurements of zeta potential, performed to evaluate the possible charge variations of the developed NPs, have not revealed the significant changes in these parameters. Taken these data together, one can conclude that the developed nanocarriers were stable for 100 days of storage at a temperature of 4°C.

The stability of the VAN-loaded-Chit-NPs-concentrate was also assessed using the optical characterization method with a TurbiScan instrument. This technique distinguishes particle movement (creaming or sedimentation) from particle size changes (flocculation or coalescence) and also provides information about the ongoing destabilization process.^30^,^31^ Figure 2b shows the Turbiscan Stability Index (TSI) with aging time. Sample was divided into three heights in the measuring cell allowing one to determine the behavior of the system in the entire volume. It is crucial to note that a lower TSI value indicates a superior stability level of the dispersion under investigation.^24^ The entire sample with different range of measuring cell was characterized by the same trend in TSI values; a clear increase followed by a flattening of the trend. Thus, the obtained TSI parameter with the aging time clearly suggested that the VAN-loaded-Chit-NPs-concentrate is stable over time, which is in line with DLS/zeta potential measurements results.

### Characterization of Hydrogels with Embedded Vancomycin-Loaded Chitosan Particles

With the systemic administration of antibiotics, it is difficult to achieve the required local delivery of medicine, and the bacteria biofilm formation is then of high risk.^9^ To address this issue, we have embedded the resulting VAN-loaded chitosan particles into biomimetic collagen/chitosan/hyaluronic acid-based hydrogels and crosslinked with genipin. Obtained in this way, composites were next freeze-dried to serve in solid flake/disc-like form, which after implantation to the target site will ensure the effective and controllable delivery of the antibiotic around the infections while reducing the potential risk of toxicity. Three concentrations of VAN-loaded chitosan particles were selected for the preparation of composites and the resulting systems (HybC1, HybC2, and HybC3) were characterized for physicochemical and biological features.

### Swelling Evaluation

**Figure 3:**
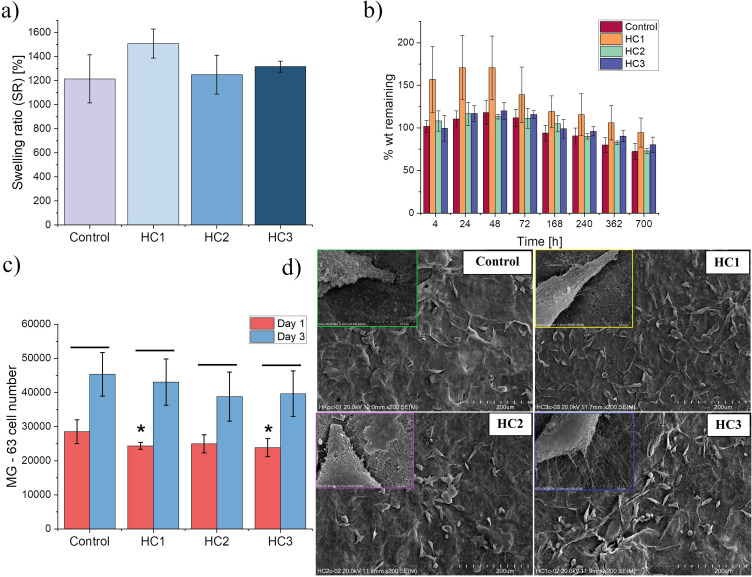
(**a**) Swelling ratio and (**b**) Degradation study under physiological conditions of prepared hydrogel systems, (**c**) The results of Alamar Blue test after 1 and 3 days of MG-63 cells culture. Statistical significance was calculated using Student’s t-test. A comparison between the two means was analyzed with a statistical significance level set at p<0.05. Below the black line indicates statistical significance between the results for the same type of material on the first and third day; *indicates statistical significance when compared with control day 1, (**d**) SEM microphotographs of MG-63 cells cultured on prepared hydrogels.

Swellability is one of the essential properties of hydrogel-based materials that strongly affects their use in drug delivery.^32^ Therefore, the developed composites were evaluated for the swelling ability under physiological conditions (PBS buffer, pH = 7.4, 37°C), and the obtained results are shown in Figure 3a. As a control, a hydrogel with embedded chitosan particles without drugs was tested. In general, the series of assessed materials did not show significant differences in the swelling factor. The highest SR value (1507.9 ± 3.6%) was observed for hydrogel with the lowest content of VAN (HC1), while the lowest SR (1213.9 ± 7.6%) was revealed for the control system. Based on the obtained results one can conclude that the swelling properties of the hydrogels did not depend on the concentration of VAN in the chitosan particles. The incorporation of particles into the hydrogel matrix can limit the mobility of the polymer chains and reduce the free volumes in the polymer network, resulting in less absorption of the aqueous solution.^33^ Furthermore, the freeze-drying process employed for the composites fabrication notably reduced the materials’ swellability since the systems of similar polymers composition presented by Gilarska et al exhibit significantly higher SR values.^21^ However, it should be emphasized that the Col/Chit/HA_mod_–based matrix still allows the hybrid materials to swell to a significant degree.

### Degradation Study

Developed hydrogel-based materials were also tested for degradation performed under physiological conditions (pH = 7.4, 37°C). The weight loss was monitored for approximately 30 days, and the results are displayed in Figure 3b. The observed tendency for all samples is similar. The kinetics of the degradation process was slow and gradual. During the first 72 hours of the experiment, weight gain was noted, and it was caused by swelling of the materials under employed conditions. A similar phenomenon of the initial increase in sample mass was presented by Guzdek–Zając et al.^34^ However, in the latter stage of the degradation study the remaining weight of all developed materials slowly decreased. After 168 hours of the degradation, a decreasing trend in mass was noticed for all samples in comparison to the initial hours of the experiment (no statistical significance). The highest mass loss after the last-point time was observed for control sample and hydrogel with a medium dose of the drug (HC2). However, no statistical significance was revealed when comparing those results to HC1 and HC3 systems. Finally, all the prepared materials after around 30 days of degradation maintained more than 70% of their initial mass. Overall, it might be concluded that VAN-loaded particles incorporated into the polymeric network do not cause faster disintegration of the resulting systems. The presented results demonstrated that the developed systems undergo slow degradation, which suggests their high cross-linking degree.

### Biological Evaluation in vitro

To verify the biocompatibility of the developed composites, we have performed preliminary biological studies in vitro utilizing MG-63 cells as a model. The results of viability tests carried out with Alamar Blue assay on the 1st and 3rd culture day are shown in Figure 3c. For all developed composites as well as the control sample (hydrogel with chitosan-based particles without loaded VAN) the analogous tendency with the prolongation of the experimental time was found. As can be noticed, the number of viable cells substantially increased after 3 days of cells culturing on the surface of all materials (statistically significant differences were revealed when compared to the results for each material at 1st and 3rd day). Comparing the 1-day result of the control sample to those for all developed composites with VAN-loaded chitosan particles, there were lower values revealed for hybrids (statistical differences revealed for HybC1 and HybC3 when compared to the control). However, after the 3rd day of culturing, a comparable number of cells were found on all tested materials. Moreover, there were no significant differences between the results obtained for VAN-loaded composites at both testing points. Overall, our findings demonstrated that vancomycin-loaded particles in the studied concentrations do not influence the rate of cells’ proliferation with respect to the control system.

Since cell adhesion is one of the important parameters in determining the biocompatibility of materials, we have also evaluated the morphology and ability of MG-63 cells to attach to the surface of developed systems. The adhesion and morphology of MG-63 cells cultured on developed materials were assessed utilizing scanning electron microscopy. Obtained SEM microphotographs are depicted in Figure 3d. As can be seen, all materials enable the adhesion of cells, which appear to be well flattened and spread. This demonstrates that the addition of VAN-loaded chitosan particles to the hydrogel matrix does not harmfully affect the cells’ adhesion. Cells on all studied surfaces have elongated shapes, which may indicate a compact and well-crosslinked structure of tested materials.^35^ This postulate is consistent with the results of the degradation experiments discussed above. Analyzing images of the higher magnifications (insets in Figure 3d), the 3D polymer network along with embedded rounded-in shaped particles is also well visible. The spherical structures exhibit a smooth surface without any signs of aggregation. We have demonstrated previously that the surface of the injectable Col/Chit/HA_mod_ hydrogels crosslinked with genipin provided a good environment for osteoblast-like cells’ adhesion.^21^ The findings revealed herein are in line with those data since composites presented within these studies promote a similar morphology of cells that are characterized by well-defined and elongated shapes. Therefore, it may be concluded that biological experiments in vitro proved the biocompatibility of the developed composites.

### Vancomycin Release Evaluation

Various mechanisms of drug release have been described previously.^36^ One of the approaches is the controlled swelling and then the balance between forces that constrain polymer network deformation and the osmosis that leads to water absorption. Another strategy is the diffusion of drugs through the matrix/delivery systems degradation. The degradation of the three-dimensional polymeric network under physiological conditions increases the size of the mesh allowing the drug to diffuse.^36^ Fluorescently labeled VAN allows the release kinetics to be tracked with greater sensitivity. Therefore, chit-NPS loaded with VAN-FITC and hydrogel systems with embedded VAN-FITC-loaded-chit-NPs were fabricated, and the antibiotic release under model physiological conditions was evaluated.

**Figure 4:**
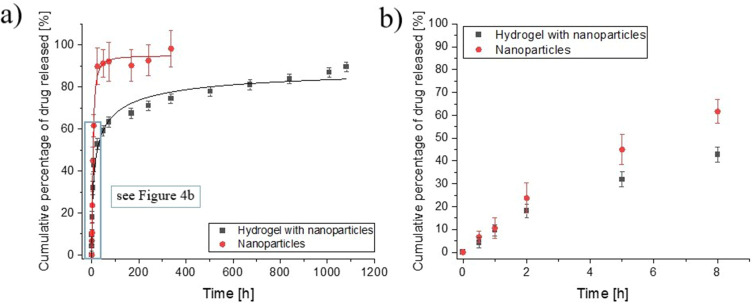
(**a**) The percentage of cumulative drug released for chit-nanoparticles loaded with VAN-FITC and hydrogel-based systems with nanoparticles embedded, respectively, (**b**) release profile during the 8-hours experiment.

The percentage of cumulative drug release from both developed formulations is presented in Figure 4a. The VAN release from nanoparticles ended after 336 hours when the cumulative percentage of drug released achieved 100%. In contrast, the hydrogels were studied for 45 days with drug release measurements taken at 30 min, 1h, 2h, 5h, 8h, 24h, 48h, 72h, 168h, 240h, 336h, 504h, 840h, 1009h, 1081h. As can be seen in Figure 4b during the first 8 hours, a significant release was noted for both systems, and following this time, a persistent release was observed. However, this process occurred substantially faster in the case of chitosan particles for which the cumulative release of VAN in 8 hours reached almost 70%. At the same time, about 40% of the medication remains within the developed hydrogel-based hybrid systems. Furthermore, after 45 days of the experiment, the cumulative percentage of drug released still does not reach 100%, indicating prolonged release of VAN (Figure 4a). These results clearly demonstrate that Col/Chit/HA_mod_-based matrix provides additional protection from uncontrolled drug diffusion.

It was previously established that the lack of antibiotic prophylaxis in surgical cases exposes the patient to a significant risk of infection.^37^ However, as reported in the literature, due to the lack of effectiveness of intravenous antibiotic therapy in the case of orthopaedic surgery, local delivery of drugs is extremely desirable.^38^,^39^ Han et al utilized a dry electrostatic powder coating technique to modified implants with vancomycin-loaded particles. These innovative solutions aim at locally delivering antibiotics to the affected tissue. Their results revealed a significant drug release during the first hours, followed by a sustained slow release.^9^ Importantly, in another study, it was found that local administration of VAN powder for spine surgeries reported no adverse effects.^37^ To sum up, our findings demonstrated that developed herein hydrogel-based systems with embedded VAN-loaded-chit-NPs could be used to reduce bacterial infections in their first acute phase and next facilitate the prolonged and graduate release.

### Antibacterial Activity Assessment

**Figure 5:**
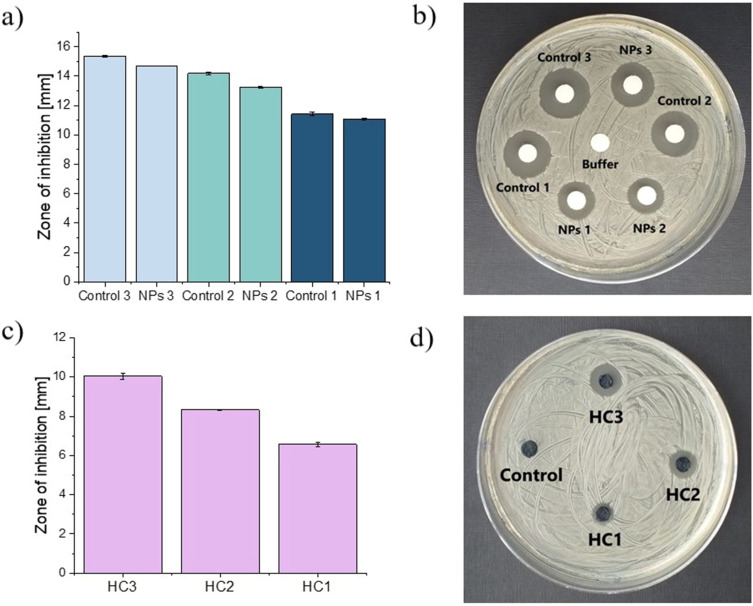
The antibacterial activity against *Staphylococcus aureus*; dependence of the inhibition zones and the representative photographs of the performed experiments for nanoparticles (**a** and **b**) and hybrid systems (**c** and **d**), respectively.

The antibacterial activity of developed systems toward *S. aureus* bacterial strain was studied as shown in Figure 5. In order to verify the expected biological activity of VAN loaded inside the chitosan particles, we have performed the antibacterial test for both developed formulations. In Figure 5a and b, the results for VAN-loaded-chit-NPs and control samples (VAN solution in acetate buffer) are depicted. The corresponding pairs of the sample (eg, NPs1 and control 1) had the same antibiotic concentration. The expected trend was revealed, namely, as the concentration of VAN increases, the zones of inhibition become larger. However, in all cases, a faintly smaller zone is observed for the NPs samples compared to the corresponding controls. Such differences might be explained assuming that under applied experimental conditions, VAN was not completely released. Overall, it was demonstrated that the vancomycin-loaded particles retain the biological activity of the antibiotic.

In the next step, hydrogel-based discs with embedded VAN-loaded-chit-NPs were tested. For all samples, the inhibition of S. *aureus* growth and proliferation was revealed. As can be seen in Figure 5c and d the bacteriostatic circle was the most pronounced for the HC3 sample with the highest VAN concentration and the lowest for HC1 hybrids. Thus, as in the case of particles, the VAN-content-dependent antibacterial activity has also been demonstrated for hybrid materials. Importantly, the inhibition zone for the hydrogel with the highest VAN content (HC3) is about 10mm, while for the NPs with the corresponding antibiotic concentration this zone is equal to about 15mm. These differences confirm that the hydrogel-based systems provide sustained drug release and are consistent with the results of kinetic studies.

## Conclusions

We have fabricated and characterized innovative composites for local VAN therapy. Developed systems are composed of chitosan particles loaded with VAN, embedded into biomimetic collagen/chitosan/hyaluronic acid-based hydrogels crosslinked with genipin and lyophilized to produce a convenient-to-use solid flake/disc-like form. The composites released antibiotics over a significantly prolonged time, minimizing the initial burst effect of vancomycin-loaded chitosan particles. Both stable over time chitosan carriers with VAN, as well as a series of materials fabricated using three concentrations of those particles, exhibited antibacterial activity against *Staphylococcus aureus*. Furthermore, the in vitro cell culture investigation demonstrated that all prepared systems’ surfaces are biocompatible since they allowed the model MG-63 cells to proliferate and adhere. Therefore, these easy-to-prepare materials have the potential to accelerate the healing process when used as implantation devices in surgical sites of different types, since besides antibacterial behavior, they can pose a site of cell attachment. Desired physicochemical features proven in swelling and degradation experiments conducted under physiological conditions confirmed the universal character of the composites, whereas a dry flake-like form ensures their long shelf life. It is expected that such a designed material will enable direct delivery of vancomycin around the infections providing its controlled/sustained release and thus will reduce the potential risk of systemic toxicity. Our data may contribute to the development of well-controllable VAN delivery systems, being a viable support or promising alternative for conventional antibiotic therapies. However, this must be verified in the extensive biological studies in vivo.

## Copyright Statement

Parts of the graphical abstract were drawn by using pictures from Servier Medical Art. Servier Medical Art by Servier is licensed under a Creative Commons Attribution 3.0 (https://creativecommons.org/licenses/by/3.0/).

## Data Sharing Statement

The datasets generated and/or analyzed during the current study are available in the RODBUK repository: https://doi.org/10.57903/UJ/MZNXTP.
